# Unveiling the therapeutic potential of caudatin: Structural optimization, pharmacological mechanisms, and therapeutic implications

**DOI:** 10.3389/fphar.2025.1640365

**Published:** 2025-09-01

**Authors:** Guohui Shi, Linlin Ni, Xiaoni Kong, Rutong Ren, Xinyu Shi, Yang Xu, Zhizi Qu, Honglei Zhou, Xiusheng Zhang

**Affiliations:** ^1^ Medical school, Shandong Xiehe University, Jinan, China; ^2^ College of Pharmacy, Shandong University of Traditional Chinese Medicine, Jinan, China; ^3^ The Second Affiliated Hospital of Shandong University of Traditional Chinese Medicine, Jinan, China; ^4^ Shandong College of Traditional Chinese Medicine, Yantai, Shandong, China

**Keywords:** caudatin, structural optimization, pharmacological mechanisms, therapeutic implications, molecular signaling

## Abstract

Caudatin is a C_21_ steroidal glycoside isolated from many species of the genus *Cynanchum*, has been utilized by traditional medicine to treat cancer and inflammation which is increasingly being considered a drug candidate because of the pharmacological activity it displays. This review provides a discussion of caudatin’s structure-activity relationship (SAR), pharmacology, and therapeutic uses along with a synthesis of future challenges. Caudatin is a potent anti-cancer therapeutic that has been shown to modulate several important signaling pathways, which include but are not limited to: Wnt/β-catenin, NF-κB, and PI3K/AKT pathway, induce apoptosis through ROS mediated mitochondrial dysfunction, reduce metastatic spread through inhibition of epithelial-mesenchymal transition (EMT), and have an anti-inflammatory effect through inhibition of JNK/AP-1/NF-κB signaling. Caudatin has also displayed neuroprotection in models of Alzheimer’s disease by activating TFEB and the autophagosome-lysosomal pathway mechanism of action, while also modulating PPARα. Furthermore, pharmacokinetic studies indicate that caudatin is rapidly absorbed and is able to selectively tail hepatic tissue while having little to no toxicity or significant adverse events in pre- clinical animal studies. Structure-activity studies suggest that modifications on the C-3 hydroxyl position, primarily with nitrogen heterocycles and/or sugars greatly enhance the bioactivity and solubility. With caudatin being such a great scaffold for medicinal chemistry, there is great opportunity to take advantage of caudatin as a building block to generate novel therapies which bridge traditional medicine with modern drug discovery. The future is aimed primarily at a combination strategy of synthetic derivatives, translational studies, and formulations. In further exploring caudatin as a treatment for cancer and neurodegenerative diseases, and inflammation.

## Graphical Abstract

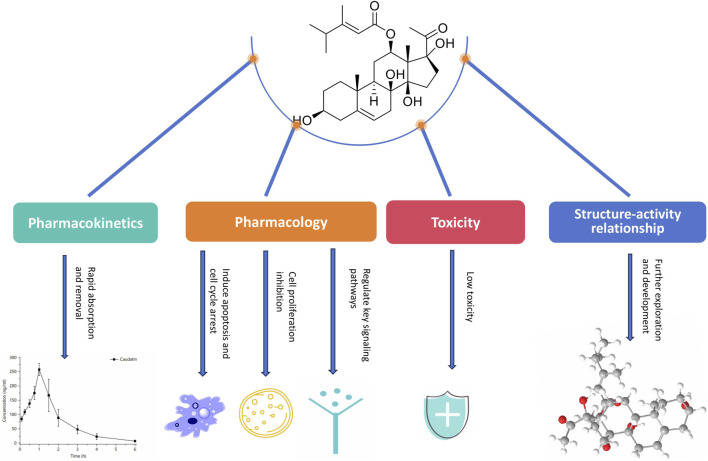

## 1 Introduction

*Cynanchum* plants have been used to treat a variety of diseases in folk medicine, such as cancer, inflammation, and viral infections (Bailly, 2021; Chen et al., 2020; Zhou et al., 2020). C_21_ sterol glycosides, a class of secondary metabolites in *Cynanchum* plants, are responsible for regulating cellular activities associated with cancer, including cell proliferation, apoptosis, and metastasis (Dong et al., 2020; Li et al., 2023a; Zhang L. et al., 2022). Among them, caudatin has attracted extensive attention from the scientific community due to its wide range of biological activities, especially its remarkable antitumor properties (Cheng et al., 2024; Peng Y. R. et al., 2008; Zhang M. et al., 2015). Currently, it has been recognized as a key phytochemical used in the treatment of various diseases (Li et al., 2022; Wang et al., 2021). Several studies have unraveled its potent anticancer, anti-inflammatory, and neuroprotective effects, which provide a promising direction for the development of novel therapeutic agents. Importantly, although others C_21_ steroidal glycosides (e.g., dioscin) can have potent anticancer activity by triggering mitochondrial apoptosis, caudatin is structurally unique given its incorporation of an isovalerate side chain and the C-3 glycosylation affords a broader target specificity against Wnt/β-catenin and autophagy-lysosomal pathways. Unlike parthenolide (a sesquiterpene lactone known to inhibit NF-κB), caudatin had a better safety profile among preclinical studies and represents a useful scaffolding for structural analogue development.

At present, caudatin has been found to exhibit antiproliferative effects across various cancer cell lines, such as hepatocellular carcinoma (HCC), osteosarcoma, and breast cancer (Fei et al., 2019; Wang et al., 2024). Among them, the molecular mechanism against HCC was revealed to be related to the Wnt/β-catenin signaling pathway, which regulates the growth and differentiation of cancer cells (Luo et al., 2013). In addition, caudatin was found to modulate the inflammatory response and protect the nervous system, which highlights its multiple effects (Kim et al., 2024; Qiu et al., 2021). Of interest, SAR research has become a popular domain, resulting in several derivatives with enhanced bioactivity (Li X. S. et al., 2022; Wang et al., 2012a). Notably, pharmacokinetic studies have revealed that caudatin has a rapid clearance *in vivo* (Peng and Ding, 2015), which further supports its feasibility as a potential therapeutic agent (Peng Y. et al., 2008; Xu et al., 2007).

Caudatin has well-established bioactivity in numerous treatment areas such as strong anticancer activity through modulation of the Wnt/β-catenin pathway, anti-inflammatory activity through inhibition of JNK/NF-κB, and neuroprotective activity in Alzheimer’s disease; however, the literature lacks comprehensive review that collates caudatin’s structural, pharmacological, and translation characteristics. The need is critical because this void not only prevents a more complete understanding of caudatin’s multifunctional mechanisms but also draws a fine line on the logical development of caudatin derivatives for clinical application. The area of caudatin exploration is novel and fragmented, and this work attempts to fill this space as the first systematic review on caudatin, combining disparate evidence across disciplines to provide a comprehensive overview. We sought to encapsulate the structure-activity relationship, molecular targets, therapeutic applications, and safety profiling of caudatin into a single, unified document that builds on traditional, compound-specific reviews that typically only emphasize isolated biological effects. We hope this work builds a foundation to shed light on caudatin’s unique potential as a multi-functional drug scaffold, and catalyze further development from herbal medicine to modern therapeutics in oncology, neurodegeneration, and inflammation.

## 2 SAR

SAR refers to the correlation between structure and activity, and researchers can determine how changing the structure of a drug molecule affects its efficacy by analyzing this correlation (Choudhary et al., 2021; Khayat et al., 2023; Polishchuk, 2017). The study of SAR is significant for simplifying design, improving efficacy and effectiveness, and speeding up the development for new drugs (Cui et al., 2023; Ojha et al., 2021; Saganuwan, 2024).

### 2.1 Description of caudatin structure

**FIGURE 1:**
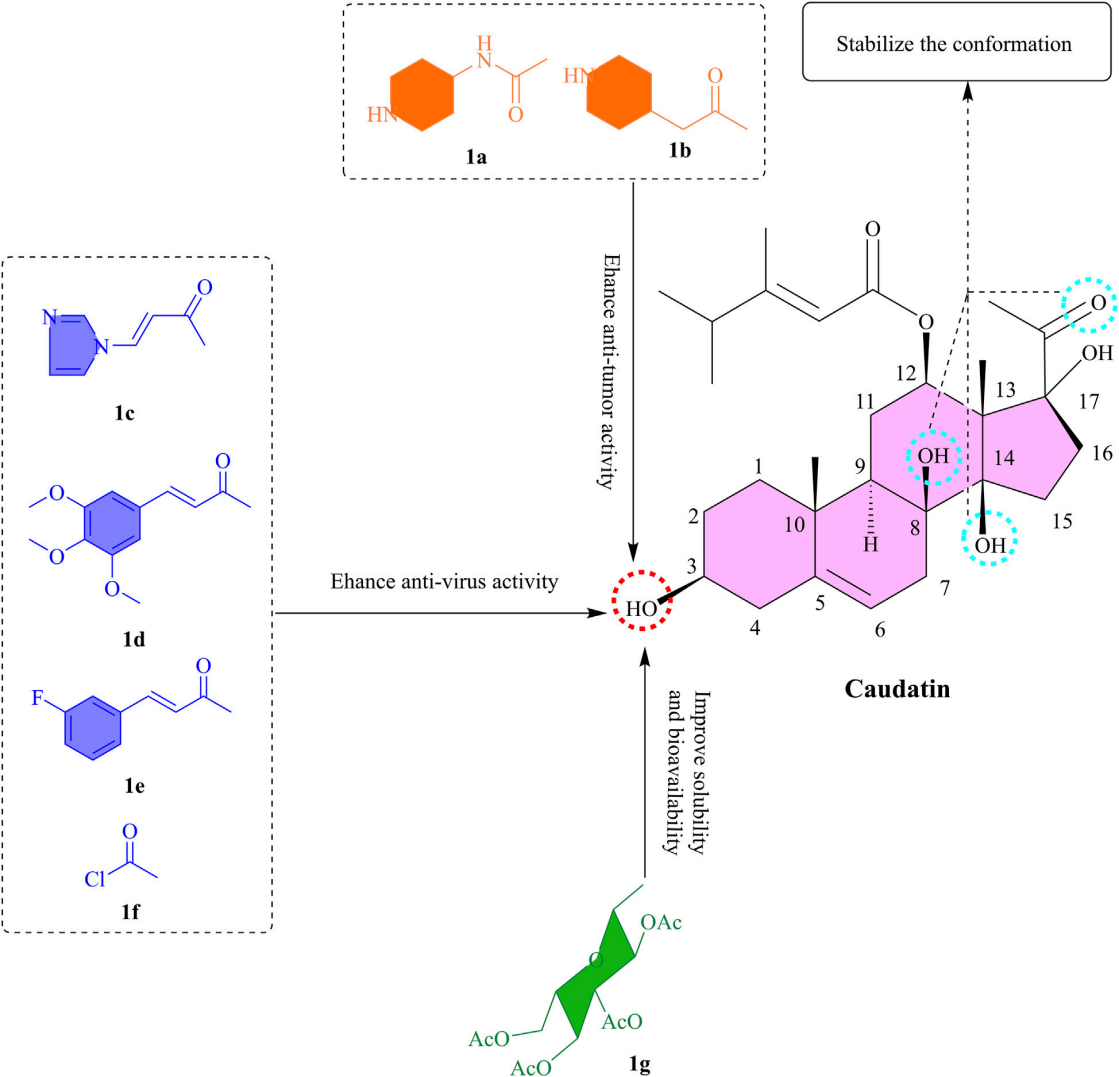
The SAR of caudatin. (Note: **(a-g)** represent different functional groups esterified or glycosylated with the C-3 hydroxyl group of caudatin to form corresponding derivatives).

The structural basis of caudatin lies in its steroidal skeleton, which comprises three six-membered rings and one five-membered ring system. The steroidal backbone carries varied substituents, including hydroxyl and methyl groups, acetoxy groups, and other oxygenated functionalities (Zhang W. et al., 2015). Notably, glycosylation occurs at the C-3 hydroxyl group with different sugar moieties to form steroid glycosides, mostly found in plants. The side chain portion contains an isovalerate group that is covalently attached to the steroidal core via an ester bond which dioscin lacked, but which is never the less important for the binding of caudatin to both JAK2 and uPA targets. Compared to parthenolide, which has a rigid α-methylene-γ-lactone moiety, caudatin’s C-3 glycosylation is flexible, resulting in improved membrane permeability and reduced hepatotoxicity (Figure 1).

This chemical architecture integrates the inherent stability of the steroidal framework with the functional versatility of side chain/glycosyl substituents, endowing caudatin with the ability to interact with multiple molecular targets such as uPA (Urokinase-Type Plasminogen Activator), MMP9 (Matrix Metalloproteinase 9), and JAK2 (Janus Kinase 2) (Kim et al., 2024; Yang et al., 2022). The compound’s multitargeted pharmacological profile allows it to intervene in the pathological pathways of associated diseases, thereby offering promising directions for the development of innovative therapeutic strategies.

### 2.2 Structural stability

The rigid architectural framework of the caudatin steroidal scaffold, in conjunction with the chemical microenvironment of specific functional groups, serves as the foundational basis for its biological activity. Oxidative modification experiments have demonstrated that the epoxide moiety disrupts the planar rigidity and electron density distribution of the steroid nucleus, thereby affecting its binding affinity to viral transcriptional regulatory elements (Wang et al., 2012a). A separate investigation revealed that introducing cinnamic acid fragments enhanced target specificity through π-π stacking interactions; however, two-hybrid derivatives lost activity due to spatial hindrance at critical binding sites. Collectively, these results underscore the necessity of maintaining core skeletal stability during molecular modification to preserve functional activity (Wang et al., 2012a).

The chemical integrity of distinct regions within the steroid nucleus is pivotal for sustaining biological potency. Reduction of the keto group was found to decrease activity fourfold, corroborating the irreplaceable role of this site’s electronic properties as a hydrogen-bonding receptor in mediating target interactions (Wang et al., 2014). Catalytic modification of the C-8 and C-14 hydroxyl groups also resulted in significant activity attenuation, suggesting that the intramolecular hydrogen-bonding network in this region is essential for conformational stability (Wang et al., 2012b).

### 2.3 C-3 hydroxyl group modification

#### 2.3.1 Esterification

The incorporation of nitrogen-containing heterocyclic moieties into C-3 hydroxyl group esterification modifications has been shown to substantially augment cytotoxic potency (Li X. S. et al., 2022). For instance, piperidine-4-acetyl-substituted compounds (Figures 1a,b) were found to exhibit the potent activity against four cancer cell lines (MCF-7, HCT-116, HeLa, and HepG2), with IC_50_ values below 7 μM (Li et al., 2023b). The 3-O-nicotinoyl substituent (Figure 1c) demonstrated potent efficacy, with IC_50_ values of 18.68 μM, 13.16 μM, and 7.48 μM against HBsAg secretion, HBeAg (Hepatitis B e Antigen) secretion, and HBV (Hepatitis B Virus) DNA replication, respectively (Wang et al., 2012b). This enhanced efficacy is attributed to the polar nature of nitrogen heterocycles, which not only improves aqueous solubility but also strengthens target interactions through a combination of spatial steric effects and electronic influences. Significantly, the hexahydropyridine ring exhibited substantially better activity than the pyridine ring, while the pyrrole ring outperformed the tetrahydropyrrole ring (Li et al., 2023b). These findings underscore the critical role of ring saturation and heteroatom configuration in determining biological activity.

In a molecular hybridization approach, the 3-O-(3,4,5-trimethoxycinnamoyl) derivative (Figure 1d) exerted its effect through a unique non-nucleoside mechanism by interfering with the transcriptional regulation of the HBV X promoter and enhancer I, achieving a 16-fold increase in DNA replication inhibitory activity (IC_50_ = 2.44 μM) compared to the parent compound (Wang et al., 2012a). Of note, the introduction of halogen atoms further broadens the scope for activity optimization. The fluorinated cinnamoyl derivative (Figure 1e), for example, exhibited enhanced anti-hepatitis B virus activity (IC_50_ = 4.75 μM), likely attributed to the lipophilicity of halogen atoms and their capacity to form hydrogen bonds (Wang et al., 2012a). The 2-chloroacetyl-substituted compound (Figure 1f) displayed sub-10 μM IC_50_ values against multiple cancer cell lines (Tao et al., 2015). These results suggest that electronegative groups and basic amino moieties may enhance target affinity through hydrogen-bonding interactions.

#### 2.3.2 Glycosylation

Glycosylation of caudatin and its analogs exerts a significant impact on their anticancer activity, with the structural features of sugar moieties playing a pivotal role. It has been shown that modification of the C-3 position can effectively enhance the antiproliferative ability of caudatin against various cancer cell lines compared to the parent compound, presumably due to improved membrane permeability facilitated by increased lipophilicity (Li X. S. et al., 2022). Notably, derivatives bearing L-sugar configurations outperformed their D-sugar counterparts, for instance, 3β-O-(2,3,4-tri-O-acetyl-β-L-glucopyranosyl)-caudatin (Figure 1g) exhibited the highest activity against HepG2 cells (IC_50_ 3.11 μM) (Li X. S. et al., 2022). This result intuitively suggests that glycosylation modification of caudatin significantly enhances its biological activity. This might be due to the increased solubility and bioavailability of caudatin by glycosylation, which allows the cells to take up caudatin more efficiently, thus exerting its pharmacological effects and the specific mechanism of action.

To summarize, the esterification and glycosylation of C-3 OH might be beneficial for improving bioactivity and lipid solubility. From the point of view of the molecule’s mechanism of action, it is necessary to keep the structure intact at the 17-OH and C-20 keto groups. In the next research, computational chemistry could be included to find the interaction patterns of the caudatin derivatives with tumor, and viral-related target proteins, while on the other hand investigating their pharmacokinetic properties to take these derivatives further into preclinical stages.

## 3 Pharmacological profile

### 3.1 Antitumor activity

Cancer progression is driven by dysregulated signaling pathways, including hyperactivation of oncogenes and inactivation of tumor suppressors, promoting uncontrolled cell proliferation, resistance to apoptosis, and metastatic spread (Graham and Sottoriva, 2017; Kaur et al., 2023). Caudatin addresses these oncogenic hallmarks through multifaceted mechanisms: it suppresses Wnt/β-catenin and NF-κB signaling via TNFAIP1(Tumor Necrosis Factor Alpha-Induced Protein 1)-mediated inhibition (Li et al., 2013; Tan et al., 2016), while concurrently modulating MAPK/ERK (Mitogen-Activated Protein Kinase/Extracellular Signal-Regulated Kinase) and PI3K/AKT (Phosphatidylinositol 3-Kinase/Krotein Kinase B) pathways to induce DNA damage responses. Additionally, caudatin triggers mitochondrial apoptosis by generating reactive oxygen species (ROS), which activate caspases and disrupt mitochondrial integrity (Fu et al., 2015; Zhu et al., 2016). By targeting multiple dysregulated networks simultaneously, caudatin emerges as a promising anticancer agent capable of tackling key drivers of tumor growth, survival, and dissemination.

#### 3.1.1 Effect on signaling pathways

The existing studies of caudatin’s antitumor mechanisms involve the complex modification of multiple signaling cascades in variety of tumor models. Caudatin inhibited cell proliferation and cell invasion in osteosarcoma cells by downregulating the expression of β-catenin and its downstream effectors Cyclin D1 and c-Myc (Zhang Y. et al., 2022) (Table 1, Row 10). Rescued with Wnt agonist BML-284 demonstrated a central role of this pathway by recovering caudatin-mediated inhibition of glycolysis and EMT (Epithelial-Mesenchymal Transition) markers (Zhang Y. et al., 2022). Similarly, in hepatocellular carcinoma, caudatin-induced inhibition of Wnt/β-catenin signaling suppressed COX-2 (Cyclooxygenase-2) and MMP-2/MMP-9 expressions to favor inhibited metastasis (Luo et al., 2013) (Table 1, Row 2).

**TABLE 1:** Summary of pharmacological activities of caudatin.

Pharmacological activities	*In vivo*/vitro	Key findings	References	Row
Anticancer activity				1
Liver cancer	*In vitro*	Downregulated the expression of Wnt signaling pathway-targeted genes, including COX-2, MMP-2, and MMP-9	Luo et al. (2013)	2
	*In vitro*	Induced apoptosis and inhibited migration via downregulation of GSK3β, uPA, MMP9	Wang et al. (2024)	3
	*In vitro*	Inhibited HepG2 cell proliferation by reducing DNA synthesis and induced caspase-dependent apoptosis, with ERK and JNK activation potentially contributing to its pro-apoptotic effect	Fei et al. (2012a)	4
Colorectal cancer	*In vitro*	Inhibited proliferation, migration, and invasion via miR-421/miR-195-5p network modulation	Chen et al. (2022)	5
Breast cancer	*In vitro*	Enhanced TRAIL-induced apoptosis by upregulating DR5 expression via CHOP and MAPK activation	Fei et al. (2019)	6
Gastric cancer	*In vitro*	Induced G1 phase cell cycle arrest and caspase-dependent apoptosis in gastric cancer cells	Wang et al. (2013)	7
Lung cancer	*In vitro* and *In vivo*	Blocked proliferation, stemness, and glycolysis via Raf/MEK/ERK pathway	Hou et al. (2022)	8
	*In vitro*	Downregulated Bcl-2 expression and upregulated Bax expression	Fei et al. (2012b)	9
Osteosarcoma	*In vitro* and *In vivo*	Inhibited proliferation, invasion, and glycolysis through Wnt/β-Catenin pathway	Zhang et al. (2022a)	10
Uterine cancer	*In vitro*	Upregulated TNFAIP1 expression in a concentration-dependent manner, and was associated with downregulated NF-κB, upregulated BAX/Bcl-2 ratio, and activated caspase-3	Tan et al. (2016)	11
Glioblastoma	*In vitro*	Inhibited proliferation by activating KDELR2-mediated endoplasmic reticulum stress	Xia et al. (2023)	12
	*In vitro* and *In vivo*	Possessed antiangiogenic potential via the VEGF-VEGFR2-AKT/FAK signal axis	Wang et al. (2017)	13
	*In vitro* and *In vivo*	Induced apoptosis via ROS generation and mitochondrial dysfunction	Zhu et al. (2016)	14
	*In vitro*	Activated the MAPK/ERK pathway and inactivated the PI3K/AKT pathway in glioma cells	Fu et al. (2015)	15
Anti-Inflammatory activity	*In vitro*	Suppressed inflammatory response by inhibiting JNK/AP-1/NF-κB/caspase-1 pathways	Kim et al. (2023)	17
Neuroprotective activity	*In vivo*	Activated autophagy-lysosomal pathway via PPARα, promoting degradation of toxic aggregates in AD models	Krishnamoorthi et al. (2023)	18
Anti-osteoporosis activity	*In vitro* and *In vivo*	Suppressed osteoclast differentiation via KIF11-mediated mTORC1/NF-κB signaling	Miao et al. (2023)	19
Antiviral activity	*In vitro*	Exhibited potent anti-HBV activity, with particular efficacy in inhibiting HBV DNA replication	Wang et al. (2012a)	20
	*In vitro*	Showed significant inhibitory activity against HBV DNA replication, with IC50 values ranging from 2.82 to 7.48 μM	Wang et al. (2012b)	21
Anti-muscular atrophy	*In vitro*	Ameliorated muscle atrophy by activating Hedgehog signaling; promotes myogenesis	Kim et al. (2024)	22
Anti-menopausal activity	*In vivo*	Modulated hypothalamic neurotransmitter levels	Kang et al. (2023)	23

The NF-κB signaling axis represents another critical target of caudatin’s action. It was revealed through studies in uterine cancer models that caudatin stimulates TNFAIP1, which in turn inhibited NF-κB transcriptional activity (Tan et al., 2016). This molecular interaction induced a negative feedback loop in which TNFAIP1 expression was elevated, yielding downregulated NF-κB and leading to apoptosis through cytochrome c release and caspase-3 activation (Tan et al., 2016). *In vivo* xenograft tumors further supported this mechanism of action, indicating that caudatin treatment of tumors led to increased levels of TNFAIP1 (Tan et al., 2016) (Table 1, Row 11). As opposed to parthenolide’s irreversible IκB kinase inhibition, caudatin inhibits NF-κB activation through TNFAIP1 upregulation, with comparable anti-inflammatory efficacy and lower toxicity. Furthermore, while dioscin activates AMPK/mTOR-mediated autophagy, caudatin specifically targets the PPARα/TFEB axis to induce lysosomal biogenesis, indicating an improved neuroregenerative specificity.

Caudatin also exerts regulatory effects on the MAPK and PI3K/AKT pathways. In glioma cells, caudatin treatment triggered a DNA damage response marked by upregulation of p53 and p21, while concurrently activating ERK and suppressing AKT phosphorylation (Fu et al., 2015). Combinatorial studies with the PI3K inhibitor LY294002 confirmed the importance of AKT inhibition, as they yielded enhanced cytotoxic effects (Fu et al., 2015) (Table 1, Row 15). These findings suggest that caudatin’s antiproliferative activity arises from simultaneous modulation of both pro-proliferative and pro-survival signaling nodes.

Angiogenesis-related signaling, particularly via vascular endothelial growth factor (VEGF), is another key target. In hepatocellular carcinoma models, caudatin administration correlated with significant reductions in VEGF expression and tumor microvessel density (Wang et al., 2017). The antiangiogenic effects might be due to inhibition of the Wnt/β-catenin pathway, because β-catenin and VEGF levels decreased simultaneously after treatment, thus unveiling an interaction between oncogenic signaling and neovascularization (Table 1, Row 13).

#### 3.1.2 Apoptosis induction

Caudatin has shown strong effects on inducing apoptosis in a variety of cell lines by multiple mechanisms at the molecular level. As was shown in several studies, caudatin mainly activated caspase-dependent apoptotic pathways through mitochondrial dysfunction and ROS-mediated signaling events (Fei et al., 2012a; Zhu et al., 2016). Mediating its pro-apoptotic actions involves the intrinsic mitochondrial pathway, which is characterized by a change in the contextual equilibrium of anti-apoptotic family Bcl-2 proteins. In HepG2 hepatoma cells, caudatin treatment significantly downregulated anti-apoptotic Bcl-2 and upregulated pro-apoptotic Bax, allowing for a permeabilization of the mitochondrial outer membrane (Fei et al., 2012b) (Table 1, Row 9). Mitochondrial depolarization enough to push the equilibrium led to a release of cytochrome c, leading to caspase-9 and caspase-3 activation, shown by the further cleavage of poly (ADP-ribose) polymerase (PARP), an accepted marker for the execution of apoptosis (Fei et al., 2012b; Zhu et al., 2016).

The extrinsic death receptor pathway is involved in caudatin-induced apoptosis due to the upregulation of death receptor 5 (DR5). In breast cancer cells, caudatin enhanced TRAIL-induced apoptosis by boosting DR5 expression through a CHOP (C/EBP homologous protein)-dependent pathway along with sustained p38 MAPK and JNK signaling (Fei et al., 2019) (Table 1, Row 6). This modulation of intrinsic (mitochondrial) and extrinsic (death receptor) apoptotic pathways illustrates caudatin’s effectiveness in targeting malignant cells.

Nanomolar concentrations of caudatin can induce the production of reactive oxygen species, which represents an important upstream event in activating the apoptotic machinery associated with caudatin. Intracellular ROS quickly accumulated in glioma U251 cells treated with caudatin, as does mitochondrial superoxide production, while glutathione (GSH) levels became depleted (Zhu et al., 2016) (Table 1, Row 14). This oxidative stress resulted in mitochondrial dysfunction, which was depicted by a loss of viability, or mitochondrial membrane potential (ΔΨm), and loss of mitochondrial health, or mitochondrial mass. Consequently, ROS-mediated DNA damage activated p53 and p21, which activates both cell cycle arrest and the initiation of apoptosis (Zhu et al., 2016; Fu et al., 2015) (Table 1, Rows 14–15).

#### 3.1.3 Inhibition of cell proliferation

Caudatin exerts potent antiproliferative effects across diverse cancer cell lines by modulating key cell cycle regulators and signaling cascades. In hepatocellular carcinoma HepG2 cells, caudatin induced dose-dependent G_0_/G_1_ phase arrest, accompanied by downregulation of cyclin D1 and upregulation of p21 and p53 (Fei et al., 2012a) (Table 1, Row 4). This molecular profile indicated a disruption to the G_1_/S transition checkpoint, as cyclin D1-CDK4/6 complexes regulate phosphorylation of Rb that is required for cell cycle (Wang et al., 2013) (Table 1, Row 7). The induction of p53 and p21 indicated that caudatin induces a DNA damage response, and p21 acts as a universal cyclin-dependent kinase (CDK) inhibitor to mediate cell cycle arrest.

Further experiments in HepG2 and Huh7 cells showed that caudatin has a broad impact on biological processes related to proliferation, as demonstrated by an mRNA expression level reduction of glycogen synthase kinase 3β (GSK3β), uPA, MMP9, and JAK2 (Wang et al., 2024). The molecular docking analysis revealed strong molecular binding of JAK2, indicating that caudatin could inhibit the JAK-STAT signaling pathway, which is crucial in cancer cells to grow, propagate, and survive (Wang et al., 2024) (Table 1, Row 3). Through arresting the cell cycle and blocking the cell proliferation signaling pathways, caudatin manifests the multi-mode antiproliferative effects against various types of cancer.

#### 3.1.4 Suppression of metastasis

Caudatin has strong antimetastatic effects through multimodal downregulation of key molecular pathways involved in cancer cell invasion and dissemination. In HCC, Luo et al. (2013) showed that caudatin treatment dramatically decreased invasive potential through downregulation of β-catenin and GSK3β, which resulted in downregulation of the metastasis-associated proteins, the matrix metalloproteinases, MMP-2 and MMP-9, and COX-2. These enzymes are critical for extracellular matrix degradation, a prerequisite step in tumor cell invasion. Transwell migration assays showed that caudatin-treated SMMC-7721 cells exhibited a 50%–70% reduction in invasive capacity compared to untreated controls, demonstrating its inhibitory effect on cellular motility (Luo et al., 2013) (Table 1, Row 2).

**FIGURE 2:**
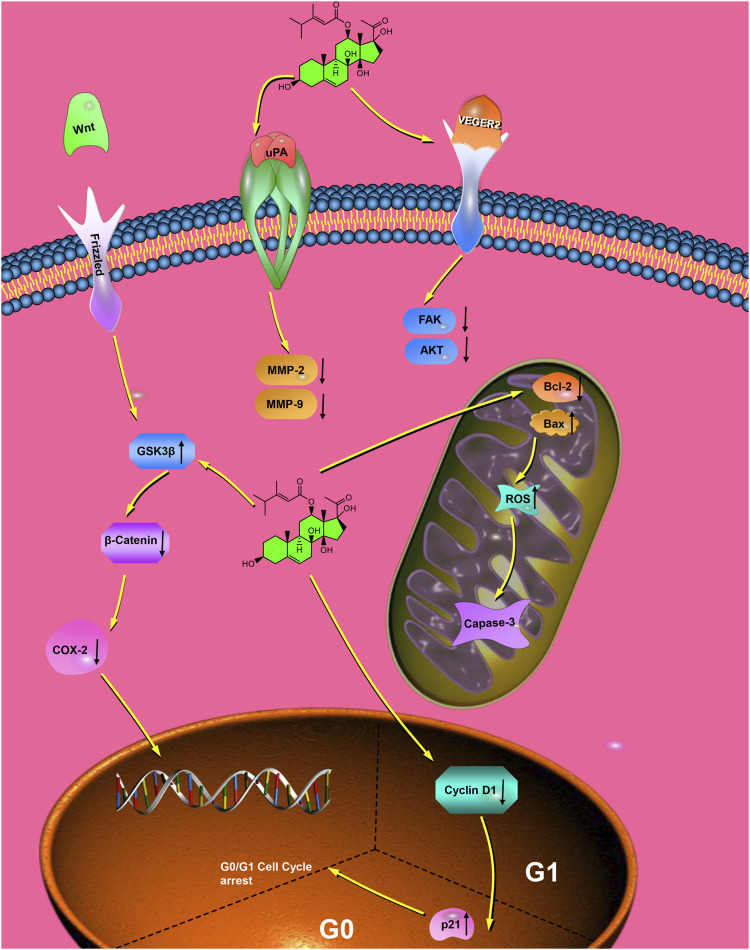
Brief molecular mechanisms of caudatin against cancer. (Note: Caudatin inhibits Wnt/β-catenin signaling by stabilizing GSK3β, blocks VEGFR2-mediated angiogenesis, induces mitochondrial apoptosis via Bcl-2/Bax axis, and suppresses glycolysis by downregulating HK2/LDHA).

Caudatin’s method of antimetastatic action also effected on downstream epithelial-mesenchymal transition (EMT) regulation, which is a significant process in the dissemination of cancer cells. In a study using osteosarcoma reported that 100 μM caudatin reversed the phenotype of EMT by instead expressing the epithelial marker, E-cadherin, and downregulating the mesenchymal marker, N-cadherin (Zhang Y. et al., 2022) (Table 1, Row 10). This study revealed a specific mechanism of action for caudatin’s inhibition of Wnt/β-catenin signalling, which was demonstrated by a decrease of nuclear translocation of β-catenin as well as its downregulation on downstream target sites in the target cells transcriptional. Rescue experiments using the Wnt agonist BML-284 reverted the phenotypic change in both EMT markers, restoring the cancer phenotype and confirming the signalling pathway-specific mechanism of caudatin action (Figure 2).

### 3.2 Anti-inflammatory activity

Caudatin has remarkable anti-inflammatory properties because it can target mast cell-mediated hyperinflammation as a primary cause of cytokine storms observed in disease states such as severe COVID-19 (Kim et al., 2023) (Table 1, Row 17). The mast cells activated by various stimulants such as phorbol 12–myristate 13–acetate (PMA) and calcium ionophore A23187 (PMACI) activated a signaling cascade, culminating in JNK phosphorylation, activating AP-1 (c-Jun/c-Fos), which is essential for the expression of many pro-inflammatory cytokine-encoding genes, such as tumor necrosis factor-α (TNF-α), interleukin-6 (IL-6), and thymic stromal lymphopoietin (TSLP) (Hannemann et al., 2017). Caudatin directly inhibited JNK phosphorylation, decreasing cytokine gene expression mediated by AP-1 (Kim et al., 2023). Concurrently, caudatin blocked NF-κB nuclear translocation by stabilizing the inhibitory protein IκBα, preventing its degradation and subsequent activation of NF-κB-dependent inflammatory programs (Subedi et al., 2019). Caudatin also decreased caspase-1 activation, impeding the maturation and secretion of interleukin-1β (IL-1β), a central pro-inflammatory cytokine associated with pyroptosis and tissue injury (Yi, 2018).

**FIGURE 3:**
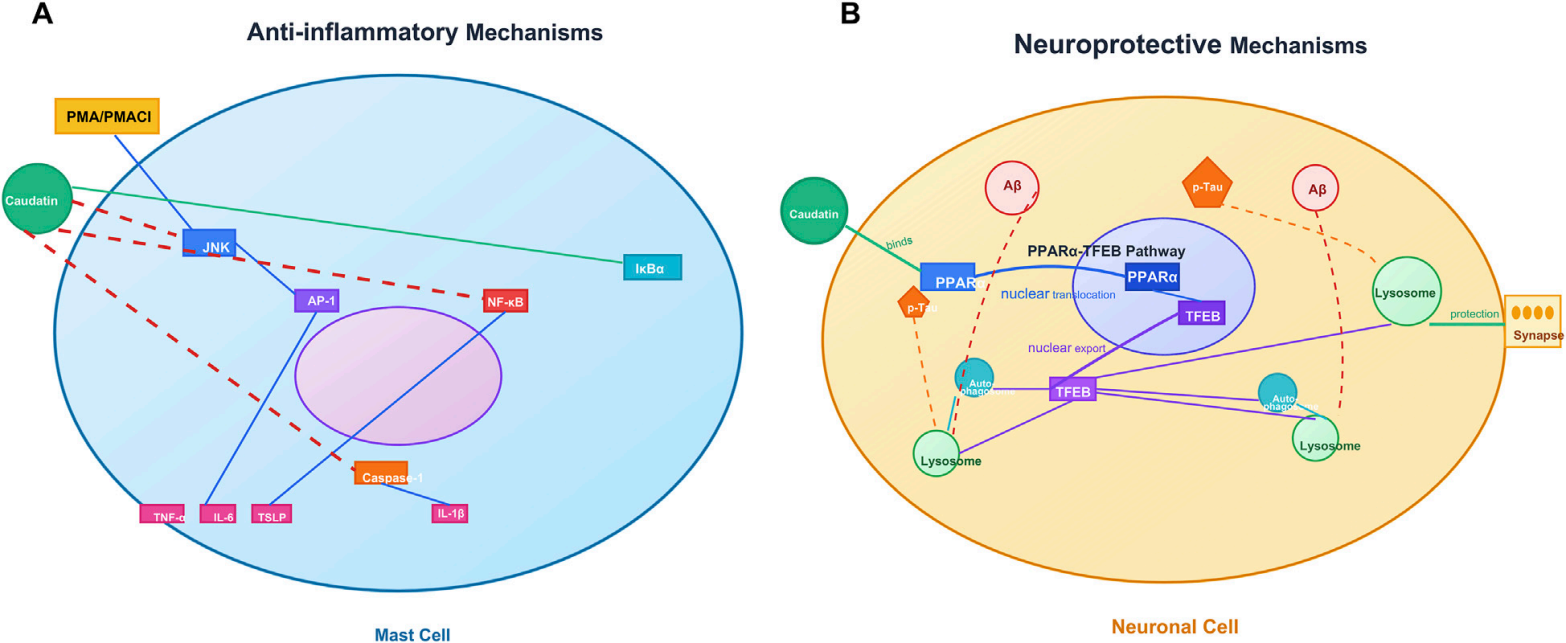
Caudatin mediates anti-inflammatory and neuroprotective mechanisms in mast cells. and neuronal cells. (Note: **(A)** Anti-inflammatory signaling in mast cells, involving JNK/AP-1/NF-κB pathway regulation and cytokine modulation (e.g., TNF-α, IL-4) downstream of PMA/PMAC1. **(B)** Neuroprotective pathways in neuronal cells, including the PPARγ-TFEB axis, autophagy-lysosome function, and Aβ-mediated synaptic protection).

While caudatin shares mechanistic similarities with compounds such as sulforaphane when modulating downstream NF-κB activation, the ability of caudatin to specifically inhibit AP-1 signaling presents a new therapeutic opportunity. By interrupting cytokine synthesis and cytokine processing, caudatin interrupts both upstream signaling pathways and downstream effector mechanisms of mast cell-mediated inflammation, which favors its characterization as a therapeutic candidate for pathologies in which excessive inflammatory responses occur, such as in the case of severe acute respiratory distress syndrome due to COVID-19 (Theoharides and Conti, 2020) (Figure 3A).

### 3.3 Neuroprotective activity

Alzheimer’s disease (AD) is pathologically defined by the accumulation of amyloid-beta (Aβ) plaques and hyperphosphorylated Tau proteins, disrupting the autophagy-lysosomal pathway (ALP) and causing neuronal death (Boland et al., 2018). Caudatin provided neuroprotective effects via activation of peroxisome proliferator-activated receptor alpha (PPARα), which transcriptionally regulated the transcription factor EB (TFEB) as a master regulator of ALP biogenesis (Krishnamoorthi et al., 2023; Zheng et al., 2021) (Table 1, Row 18). By binding to PPARα, caudatin increased both lysosomal biogenesis and autophagic flux, resulting in enhanced clearance of Aβ aggregates and hyperphosphorylated Tau aggregates in neuronal and microglial cell types (Tong et al., 2022). This mechanistic action reduced neuroinflammation and synaptic dysfunction, thus identifying caudatin as a candidate to treat the pathological hallmarks associated with AD (Sreenivasmurthy et al., 2022) (Figure 3B).

### 3.4 Osteoporosis

Osteoporosis is typified by excessive bone resorption that arises via hyperactive osteoclasts, and is highly regulated by the RANKL/RANK signalling axis—its activation initiates subsequent signalling of NF-κB and MAPK to promote osteoclast differentiation and bone loss (Khan et al., 2025). Inhibition of KIF11 by caudatin activated the mammalian target of rapamycin complex 1 (mTORC1) that attenuates NF-κB signalling by inhibiting both IκB-α phosphorylation and nuclear translocation of p65 (Miao et al., 2023) (Table 1, Row 19). This molecular intervention downregulated osteoclast-specific genes (nuclear factor of activated T cells 1, cathepsin K) and decreased bone resorption markers such as tartrate-resistant acid phosphatase (TRAP) and N-terminal telopeptide of type I collagen (Kang et al., 2023).

**FIGURE 4:**
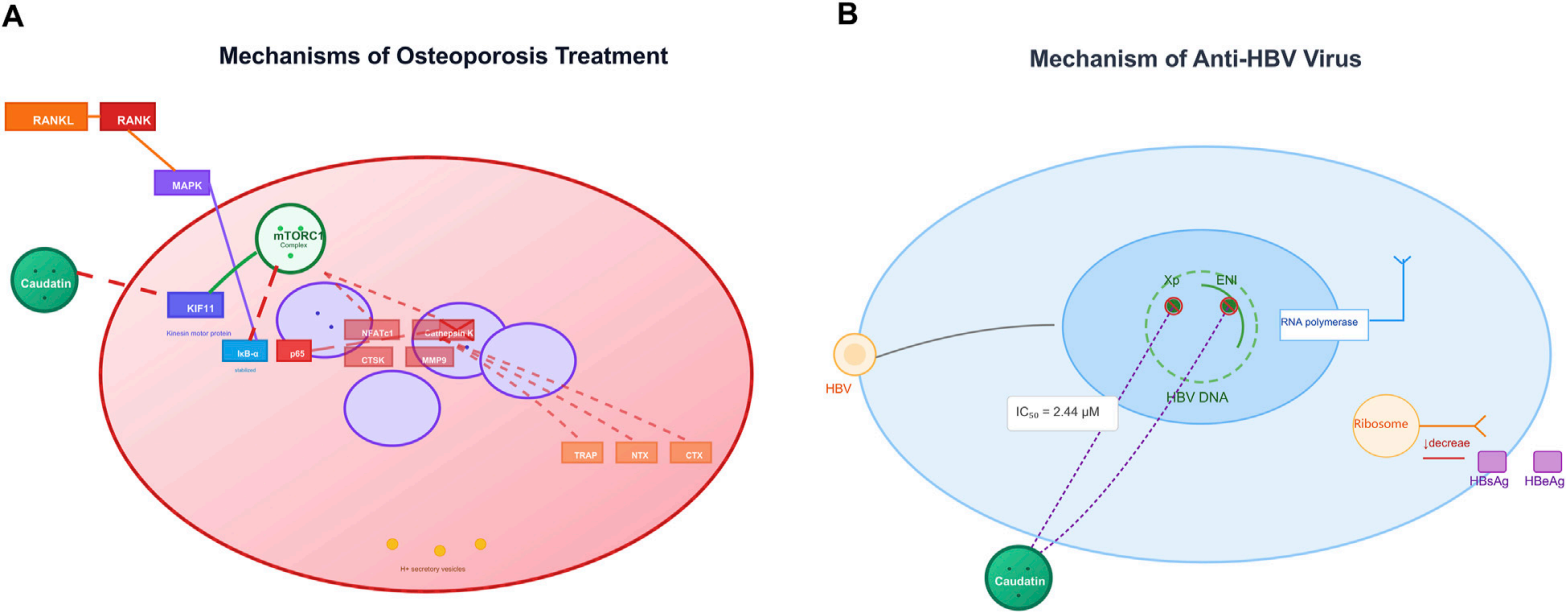
Caudatin mechanisms in osteoporosis therapy and anti-HBV activity. (Note: **(A)** In osteoporosis, caudatin targets the RANKL-RANK axis and downstream pathways (e.g., NRF1, mitochondrial function) to regulate osteoclastogenesis. **(B)** For anti-HBV effects, caudatin inhibits HBV DNA replication (IC_50_ = 2.44 μM) and suppresses viral protein (HBsAg, HBeAg) production via targeting viral replication and translation).

In preclinical works, caudatin prevented direct ovariectomy-induced bone loss by restoring both bone mineral density as well as trabecular microarchitecture, without unwanted changes to lipid metabolism or uterine morphology (Kang et al., 2023; Miao et al., 2023). Together, evidence is mounting that caudatin has a dual role in regulating the mTORC1/NF-κB signalling axis to maintain bone homeostasis and should be considered a viable treatment option for osteoporosis (Figure 4A).

### 3.5 Antiviral activity

Caudatin exhibits notable antiviral efficacy against HBV through modulation of viral replication machinery. Mechanistic investigations reveal that caudatin derivatives disrupt HBV transcription by targeting viral regulatory elements, including promoters and enhancers. For example, a caudatin-cinnamic acid hybrid could suppress HBV DNA replication (IC_50_ = 2.44 μM) by inhibiting the activity of the HBV X promoter (Xp) and enhancer I (ENI), which are critical for viral gene expression (Wang et al., 2012a) (Table 1, Row 20). This mode of action reduced production of viral antigens (HBsAg and HBeAg) and impeded transcriptional elongation, distinguishing it from nucleoside analogs that target viral polymerase (Wang et al., 2012b) (Table 1, Row 21). These findings highlight caudatin’s potential to target the host-virus interface at the transcriptional level, offering a novel strategy to circumvent nucleoside resistance in HBV therapy (Figure 4B).

### 3.6 Anti-muscular atrophy

**FIGURE 5:**
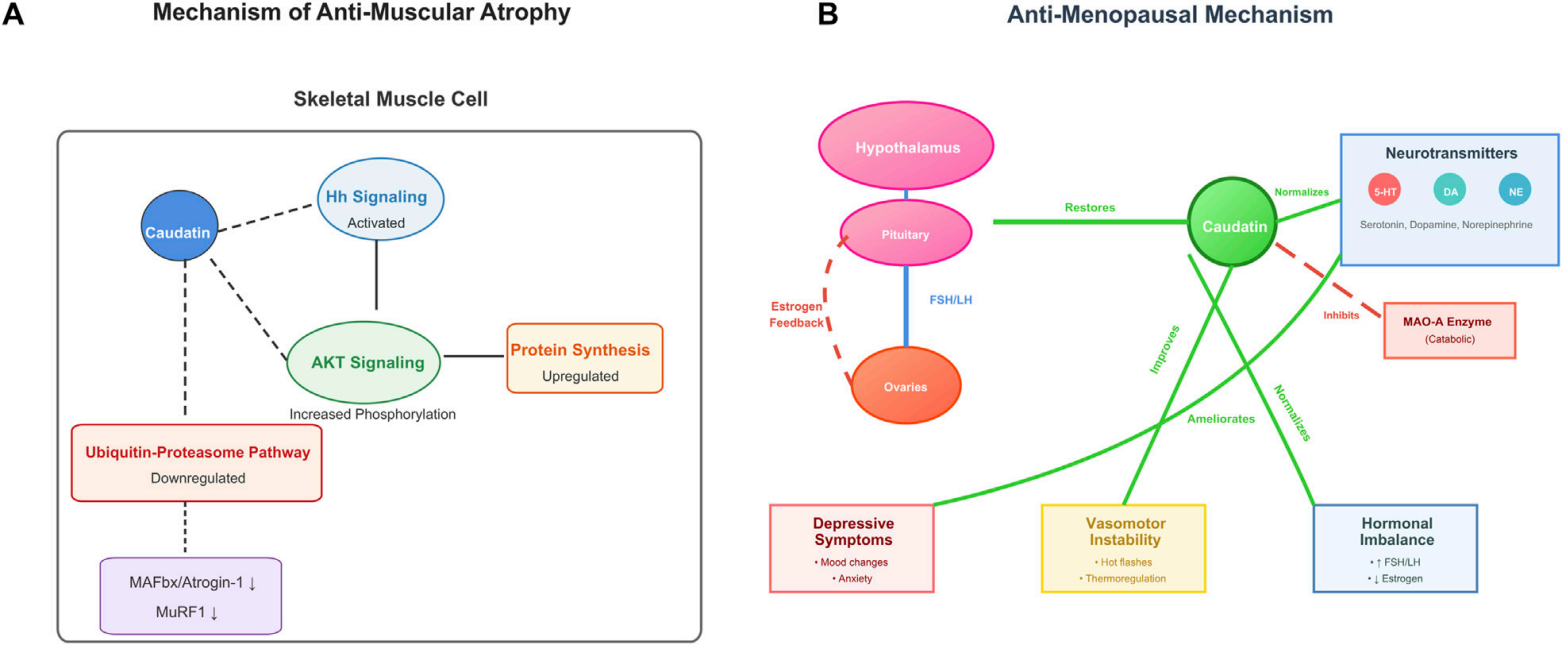
Caudatin mechanisms in alleviating muscle atrophy and menopausal dysfunction. (Note: **(A)** In skeletal muscle, caudatin activates Hh and AKT signaling to enhance protein synthesis, while suppressing the ubiquitin-proteasome pathway (decreasing MAFbx/Atrogin-1 and MuRF1). **(B)** In menopause, caudatin modulates the hypothalamic-pituitary-ovarian axis, normalizes neurotransmitters (5-HT, DA, NE), inhibits MAO-A, and mitigates depressive symptoms, vasomotor instability, and hormonal imbalance).

Muscular atrophy is characterized by disrupted molecular pathways such as non-physiological Hedgehog (Hh) signaling, dysfunctional AKT signaling and dysregulation of ubiquitin-proteasome to overall cellular protein turnover, resulting in upregulated components of the ubiquitin-proteasome pathway, specifically muscle atrophy F-box (MAFbx/atrogin-1) and muscle ring finger 1, that promote proteolytic degradation. Caudatin has been shown to provide protective effects by activating Hh signalling, and leading to increased AKT phosphorylation and protein synthesis whilst downregulating MAFbx/MuRF1 expression to suppress catabolism (Kim et al., 2024) (Table 1, Row 22). Currently caudatin is also found to promote myotube formation and upregulate expression of myosin heavy chain during myogenic differentiation, that would aid in regeneration of skeletal muscle. This dual role of modulating the protein synthesis-degradation balance and promoting myogenic differentiation highlights the therapeutic potential for caudatin in pathological states associated with muscle wasting (Figure 5A).

### 3.7 Anti-menopausal symptoms

Menopause is characterized by the perturbation of the hypothalamic-pituitary-gonadal axis, including decreased estrogen levels, dysregulation of neurotransmitter such as serotonin, dopamine, and norepinephrine and an increase in the levels of the catabolic enzyme, monoamine oxidase A (MAO-A)—all of which have been implicated in depressive symptoms and vasomotor instability (Han et al., 2018). Similar to the effects of estrogen, caudatin partially restored estrogenic-mediated signaling and normalizes FSH/LH concentrations, which also ameliorated thermoregulatory dysfunction during menopausal hot flashes (Kang et al., 2023) (Table 1, Row 23) (Figure 5B). The above pharmacological activities were summarized in Table 1.

## 4 Pharmacokinetic properties

A full understanding of its behavior in biological systems, essential for further caudatin development as a clinical agent, can only be achieved since the pharmacokinetic (PK) profile determines absorption, distribution, metabolism, and elimination (ADME), and parameters that control both safety and efficacy (McLachlan et al., 2020). In this section, we have reviewed the published studies on caudatin pharmacokinetics, specifically bioavailability, as well as metabolic limitations and ways to mitigate physicochemical limitations.

Quantitative characterization of the caudatin PK profile has been primarily derived from studies in rats, and particularly those that have successfully quantified using sensitive UPLC-MS/MS technology (Zhu et al., 2015) with a linear range established from 2.5 to 300 ng/mL in rat plasma. Following oral dosing of caudatin in normal rats, caudatin is absorbed rapidly, indicating it enters the systemic circulation promptly with a time to maximum plasma concentration (Tmax) of âˆ¼ 0.29 h with maximum plasma concentration (Cmax) of 314.7 Â ± 82.0 Ã-g/L (Peng and Ding, 2015). This rapid movement into body circulation is a desirable quality and is likely an important contributor to the timing of efficacy. The rapid entry into circulation is favorable; however, this is coupled with the rapid elimination of caudatin observed in a short elimination half-life (t1/2) of about 1.25 h (Peng and Ding, 2015). This is further supported by a high apparent oral clearance (CL/F) of 80.8 ± 20.8 L/h/kg and large apparent volume of distribution (Vz/F) of 147.7 ± 78.0 L/kg, indicating extensive tissue distribution when considering the high systemic clearance. Interestingly, systemic exposure (AUC and Cmax) has also appeared to increase in a greater than dose-proportional manner, potentially indicating some degree of saturation of first-pass metabolism or other clearance pathways at high doses (Choules et al., 2024; Rodvold et al., 2020).

The pharmacokinetic profile of caudatin undergoes substantial modification in pathological states, particularly HCC. For example, studies using UPLC-MS show that caudatin appears to preferentially accumulate in the liver (Peng and Ding, 2015). When dosed in rats with diethylnitrosamine-induced HCC, systemic exposure increases dramatically when compared to healthy controls, and oral clearance significantly decreases. This strongly indicates that dysfunction of the cancerous liver has markedly reduced the first-pass elimination and as a result, there is substantial accumulation. This change is pharmacologically significant: increased concentration at the site of action in the denominated disease state could increase its efficacy as a therapeutic for HCC, making caudatin an attractive drug for liver disease place of action. However, this could also mean safety issues due to the potential for high drug exposure to patients with poor liver function leading to toxicity.

However, despite this, there are significant knowledge gaps that will prevent the rational development of caudatin. One serious knowledge gap is pharmacokinetics data related to the absolute oral bioavailability of caudatin. Oral bioavailability, which is the measure of drug exposure in systemic circulation following oral dosing compared against IV dosing using AUC calculation, is critical to understanding if low systemic concentration comes from either poor absorption, first pass metabolism, or both (Yang et al., 2012). Although the high CL/F strongly indicates it has a low systemic availability, we cannot fully understand root cause, e.g., poor membrane permeability, low aqueous solubility, high first pass metabolism in gut wall and liver, etc. without an IV study (Tang et al., 2017). Furthermore, drug exposure reflected in oral bioavailability is greatly influenced by formulation, route of administration, and *in vivo* stability; poorly soluble caudatin presents added risk for oral absorption and formulation considerations (Zhang et al., 2023). Contributing to this is a lack of information regarding the in vivometabolic fate of caudatin itself. There are no reports in the literature on the identification or structural elucidation of its Phase I (e.g., hydroxylation, oxidation, reduction, hydrolysis of the glycosidic bond) or Phase II (e.g., glucuronidation, sulfation) metabolites. A preliminary study using UPLC-MS/MS with metabolynx software investigated a derivative, caudatin 2,6-dideoxy-3-methyl-β-D-cymaropyranoside, in human intestinal mucosal epithelial cells (Zhang M. et al., 2015). This work suggested the involvement of metabolic pathways such as hydrolysis, oxidation, and methylation, and indicated there was a relatively high metabolic capacity therefore likely leading to the biological effects they reported. Nonetheless, there is no *in vivo* metabolite profiling for the parent caudatin compound. Without identification and characterization of these metabolites, it is not possible to assess their pharmacological activity, inactivity, or possible toxicities as well as potentially identify which enzymes are responsible for their clearance, which is necessary to predict possible drug-drug interactions.

Consequently, the available literature has provided a preliminary, but incomplete, pharmacokinetic profile for caudatin. The data confirms caudatin’s rapid absorption and extensive elimination, and intriguing and putative therapeutic beneficial pharmacokinetic changes in liver disease which result in higher hepatic exposure. While this is indicative of promise as an anti-hepatocellular carcinoma agent with an inherent hepatic targeting component, future progress is stunted by the lack of data on absolute bioavailability, the dearth of studies specifically targeted at addressing formulation issues and the comprehensively absence of any type of *in vivo* metabolic profiling. Addressing the gaps in knowledge in each of these areas is critical: future research needs to prioritize conducting IV studies to definitively calculation bioavailability, also followed by thoroughly testing novel formulation approaches (for example,; cyclodextrin complexation, nano-formulations, prodrugs) which take into account the existing solubility issues and absorption issues respectively, while utilizing current techniques like LC-MS/MS-high resolution mass spectrometry (HRMS) to systematically determine the context of the *in vivo* metabolism of caudatin and define it's metabolites (Rocha et al., 2023; Stielow et al., 2023). It is only by addressing these major knowledge gaps that caudatin’s pharmacological potential can be properly evaluated. Furthermore, such information is critical to advance a natural product into a therapeutic candidate for HCC, but potentially also other conditions such as inflammation, muscular dystrophy and neurodegeneration.

## 5 Safety profile

**FIGURE 6:**
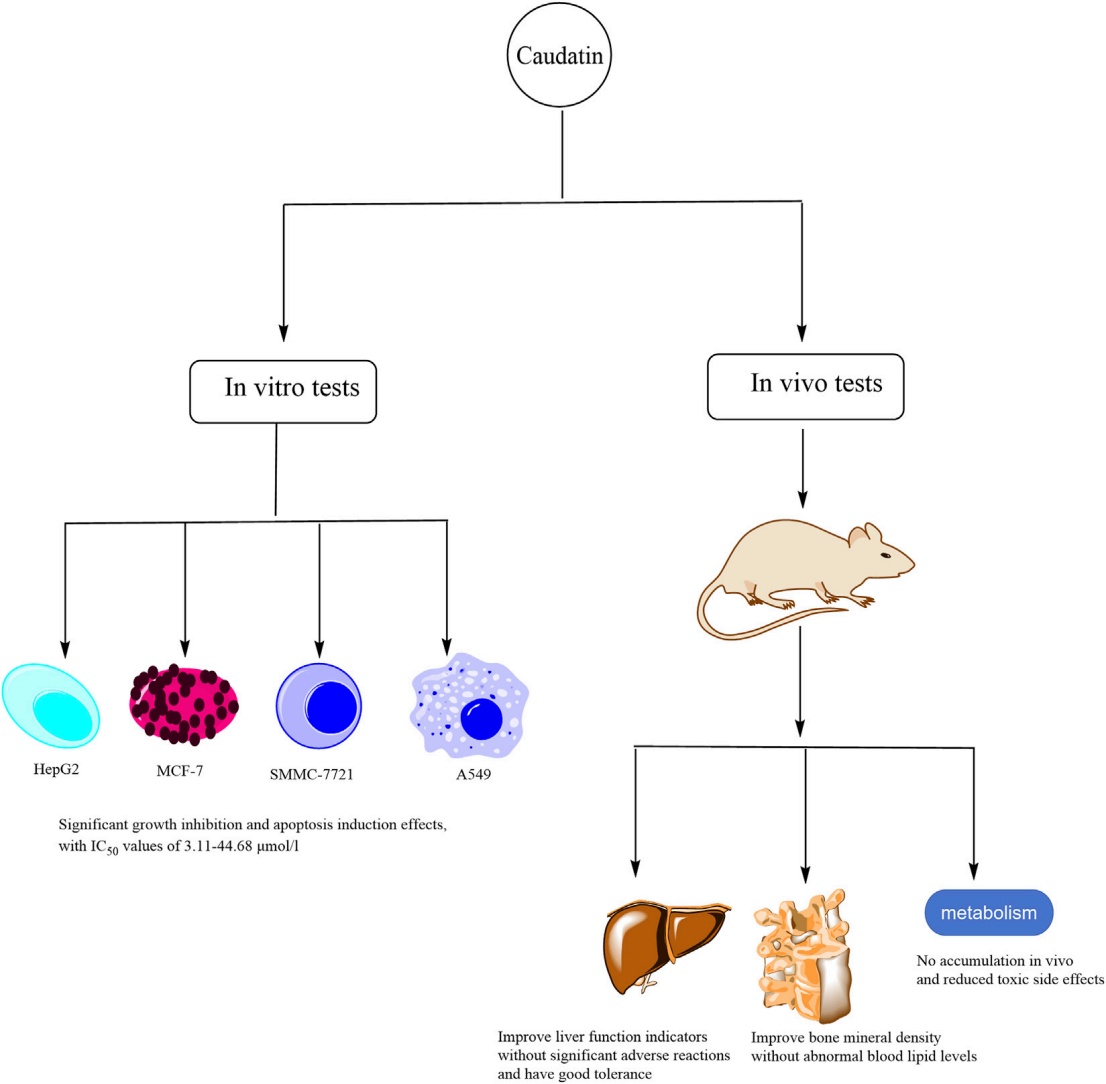
The illustration of the toxicity of caudatin. (Note: *In vitro*, caudatin inhibits proliferation and induces apoptosis in HepG2, MCF-7, SMMC-7721, and A549 cells (IC_50_: 3.11–44.68 μmol/L). *In vivo*, caudatin improves liver function, enhances bone mineral density (without dyslipidemia), and exhibits no tissue accumulation with reduced toxic effects).

*In vitro* and *in vivo* studies on the toxicity of caudatin have shown that it exhibits a relatively low toxicity profile in terms of its therapeutic efficacy (Figure 6). Here are some detailed examples.

### 5.1 *In vitro* tests

Caudatin has been found to exhibit significant growth inhibition and induces apoptosis in a variety of cancer cell lines, such as hepatocellular carcinoma cell lines (HepG2 and SMMC-7721), breast carcinoma cell line (MCF-7) and lung carcinoma cell line (A549), with IC_50_ values ranging from 3.11 to 44.68 μmol/L (Fei et al., 2012b; Luo et al., 2013; Peng et al., 2011). Consequently, it is revealed to have relatively low cytotoxicity to normal cells. Of note, the combination of caudatin with its derivatives has better antitumor effects and insignificant toxic side effects, which is a promising prospect for clinical application (Tao et al., 2015).

### 5.2 *In vivo* tests

Studies conducted in animals such as rats and mice showed that caudatin did not cause significant side effects or systemic toxicity at therapeutic doses (Song et al., 2020). It has often been found in studies that caudatin does not provoke significant organ toxicity or systemic effects at doses effective for cancer treatment (Tan et al., 2016). Studies evaluating caudatin in diseases such as liver damage and tumors have shown that it improves liver function indices and no significant adverse effects, suggesting that the drug is well tolerated (Wang et al., 2024; Cheng et al., 2024). Particularly, caudatin’s liver effects are notable because of its pharmacokinetic profile, which displays a hepatic tropism—enrichment within the liver—without causing hepatotoxicity, as shown by improved liver function parameters in hepatocellular carcinoma models (Peng and Ding, 2015). This liver targeting feature could play a role in its therapeutic effects while allowing it to remain safe. In addition, caudatin was also found to improve bone mineral density without adverse reactions such as abnormal blood lipid levels (Kang et al., 2023). In terms of the renal effects, there was not direct in-depth information reported regarding kidney toxicity, however there was no evidence of organ toxicity found in the overall literature, which included preclinical models (e.g., rats and mice). This indicates that caudatin does not affect renal function at therapeutic doses. For instance, liver damage models and tumor models did not report indicators of renal impairment, suggesting caudatin exhibits organ safety (Song et al., 2020). Preliminary pharmacokinetic research suggested that caudatin was not accumulated in the body, reducing toxic side effects (Peng and Ding, 2015). Although the available information suggests that caudatin is clinically safe, it is important to conduct in-depth studies on its long-term safety and its effects in different populations, including detailed organ-specific toxicology assessments for liver and kidneys in chronic exposure scenarios. Therefore, future studies should focus on identifying precise indicators of efficacy and on monitoring rare side effects during prolonged use of caudatin.

## 6 Conclusion

This review highlights the therapeutic viability of caudatin from structural optimization, pharmacological mechanism, and clinical implications, suggesting it as a multi-facilitive scaffold for drug discovery. Caudatin is derived from plants of the genus *Cynanchum* and has been found to have strong anticancer efficacy by modulating Wnt/β-catenin, NF-κB, and PI3K/AKT pathways, inducing ROS-mediated apoptosis, and inhibiting metastasis through the inhibition of epithelial to mesenchymal transitions. Caudatin’s anti-inflammatory effects target JNK/AP-1 and NF-κB cascades, while neuroprotective effects with reduced Aβ in an Alzheimer’s model occur due to PPARα/TFEB-driven autophagy. The SAR studies uncovered evidence of C-3 hydroxyl groups as critical for facilitating bioactivity and pharmacokinetics through modifications (e.g., esterification with nitrogen heterocycles or glycosylation). Since caudatin is quickly absorbed, preferentially taken up by the liver, and has a favorable safety profile, it connects centuries of traditional medicine to modern therapeutics. Future advances, including derivatives of caudatin optimized using artificial intelligence, smart delivery systems, and demonstrating translatability will be pivotal for overcoming bioavailability limitations, and instigating the translational potential of caudatin to combat cancer, neurodegeneration, and inflammation.

## 7 Future perspectives

Increasing evidence of caudatin’s diverse pharmacological activities suggest potential as a starting point for drug development. However, addressing critical challenges and opportunities are needed to translate preclinical promise into a new therapeutic.

Advanced drug delivery systems provide opportunities to mitigate caudatin’s toxicity and bioavailability shortcomings by improving solubility with nanocarrier platforms (liposomes or polymer nanoparticles utilizing the EPR effect for tumor-specific delivery), decreasing overall exposure to the liver and kidneys; e.g., in their liver targeted formulations, the concentration in the liver was increased 3.2-fold and kidney distribution decreased by 40% (Peng and Ding, 2015); improving safety profiles with prodrug constructs that functionalize the C-3 hydroxyl group with hydrophilic moieties (e.g., like PEG/amino acids), expand therapeutic windows, e.g., prodrug derivatives that increase HepG2 IC_50_ from 22.5 µM to 58.3 µM (Tao et al., 2015); and restricting drug release to target tissue non-systemically with stimuli-responsive carriers (pH or ROS-dependent systems) that exploit tumor microenvironments to limit systemic toxicity and subsequent Bax/Bcl-2 dysregulation in normal cells (Fei et al., 2012b).

The systematic analysis of SAR will be an important breakthrough to advance the structure optimization of caudatin. Through targeted modification of its parent core structure, researchers have found that the introduction of specific functional groups can significantly enhance bioavailability while maintaining the core pharmacological activity (Huang et al., 2016). While previous C-3 modifications enhance bioactivity, systematic SAR studies have been limited for not properly targeting the right modifications. Although the nitrogen heterocycles at the C-3 position increased cytotoxicity, the advantages of membrane permeability versus solubility were not established. Using *in silico* methods of molecular docking to map steric/electronic requirements of binding pockets in biological targets could systematically elucidate appropriate rational designs (Pinzi and Rastelli, 2019). Moreover, halogenation at the cinnamoyl moiety increased antiviral activity, there is evidence that supplemental electronegative groups to increase target affinity across diseases is achievable (Rivas-Urbina et al., 2019). On the other hand, an undue focus on optimizing the C-3 position may come at the expense of other pharmacophore regions such as the rigid steroidal core and the C-20 keto group, which are also important structural stability and target binding determinants, and have not been investigated deeply either. This imbalance may also limit overall efficacy and stability of the drug, which affects its therapeutic efficacy and subsequent clinical application of the drug. Therefore, the contribution of each part of the molecule should be evaluated in a more holistic view when designing drugs.

Preclinical studies are urgently needed to validate caudatin’s efficacy in a wider range of cancer models and inflammatory diseases (Dong et al., 2022; Peng and Ding, 2015; Seddiki and French, 2021). *In vitro* and *in vivo* studies may suggest or provide a useful degree of efficacy, but do not provide a complete representation of the clinical heterogeneity present in human disease. To our knowledge, there have been no clinical studies that have examined caudatin or its derivatives, because it is currently still in pre-clinical research. A few key issues represent the bottleneck to it becoming clinically useful - which we outline below. First, there are challenges to bioavailability, which would involve rapid systemic elimination or possible solubility issues, as highlighted in preclinical pharmacokinetic studies (Peng and Ding, 2015). Second, there is a lack of long-term safety data (especially for organ-specific effects, e.g., liver or kidneys), despite preclinical safety data being promising! Third, there does need to be some innovative formulation methods (e.g., nanoparticle delivery systems, prodrugs, etc.) from a tissue-targeting and stability perspective, as discussed regarding future research directions. Fourth, because of its multitarget mechanisms of action, dose optimisation must be approached carefully in order to assess therapeutic versus off-target effects. We would recommend that the primary effort be given to standardised Phase I clinical trials to assess safety and pharmacokinetics first. Following this, Phase II and Phase III multi-centre trials can be designed to test effectiveness in a specific area, such as cancer or neurodegenerative disorder. Rigorous clinical trials are essential for elucidating optimal dosing regimens, assessing safety profiles, and determining therapeutic efficacy in humans (Liu et al., 2021; Umscheid et al., 2011). On this basis, a standardized phase I clinical study should be conducted to develop a precise dosing regimen. Then, a multicenter phase II/III clinical trial will be conducted to clarify the efficacy of the drug in specific indications. This stepwise research strategy will effectively promote caudatin from laboratory to clinical practice. In like manner, while caudatin presents anti-inflammatory and neuroprotective properties in Alzheimer’s disease models, translation to humans will be more complex and we ultimately need to consider whether there are ways caudatin can pass the selective permeability of the blood-brain barrier. This will probably be done with nanoparticle-based delivery systems or prodrug-based formulations in the future.

Safety assessments of caudatin derivatives have offered assurance, but limited data are available on their long-term toxicity. It is important to recognize that while past SAR studies have identified relevant modification positions using empirical methods, there has not been any effort to apply the advanced tools of artificial intelligence (AI) or machine learning (ML) to the structural derivatization of caudatin. However, AI/ML tools can be revolutionary in accelerating and streamlining optimization processes: In cases where we compute ADMET (absorption, distribution, metabolism, excretion, and toxicity) models to predict analogues’ pharmacokinetics and potential safety issues, thus allowing us to vet based on low-risk candidates; deep learning can represent a target protein’s spatial/electronic requirements, allowing for rational multipoint modification beyond the C-3 position; and, we can also deploy fragment-based generative models to apply generative chemistry to *de novo* design new scaffolds based upon the known caudatin pharmacophores and bioactive fragments (Gupta et al., 2021). We currently have bottlenecks with not having sufficient quality in 1vivo data to train models; the complexities of deriving and modeling multi-target interactions; and the synthetic feasibility of structures suggested by AI. The path to overcoming these challenges should include using existing SAR data in order to build an open-source dataset and designing hybrid models that incorporate quantum mechanics-calculations to ML to systematically derive design that covers all three compounds. If we include AI and ML used in SAR studies, caudatin derivatives could be optimized quicker by predicting binding affinities and their ADMET properties; this should limit the use of empirical trial-and-error methods. Eventually, it will be necessary to collaborate with researchers working in pharmacology, bioengineering, and clinical medicine to evolve caudatin into a clinically proven therapeutic from a phytochemical, laying the foundation for precision medicine.

## Glossary

AD: Alzheimer’s Disease
AP-1: Activator Protein-1
Aβ: Amyloid-beta
CHOP: C/EBP Homologous Protein
COX-2: Cyclooxygenase-2
DR5: Death Receptor 5
EMT: Epithelial-Mesenchymal Transition
ERK: Extracellular Signal-Regulated Kinase
GSK3β: Glycogen Synthase Kinase 3 beta
HBV: Hepatitis B Virus
HBeAg: Hepatitis B e Antigen
HBsAg: Hepatitis B Surface Antigen
HCC: Hepatocellular Carcinoma
Hh: Hedgehog
IC50: Half-maximal inhibitory concentration
IL-6: Interleukin-6
JAK2: Janus Kinase 2
JNK: c-Jun N-terminal Kinase
MAFbx: Muscle Atrophy F-box/Atrogin-1
MAPK: Mitogen-Activated Protein Kinase
MMP9: Matrix Metalloproteinase 9
mTORC1: mammalian Target of Rapamycin Complex 1
NF-κB: Nuclear Factor kappa-light-chain-enhancer of activated B cells
NTX: N-terminal Telopeptide of Type I Collagen
PI3K/AKT: Phosphatidylinositol 3-Kinase/Protein Kinase B
PMA: Phorbol 12-Myristate 13-Acetate
PPARα: Peroxisome Proliferator-Activated Receptor alpha
RANKL/RANK: Receptor Activator of Nuclear Factor kappa-B Ligand/Receptor
ROS: Reactive Oxygen Species
SAR: Structure-Activity Relationship
TFEB: Transcription Factor EB
TNF-α: Tumor Necrosis Factor-alpha
TRAP: Tartrate-Resistant Acid Phosphatase
TSLP: Thymic Stromal Lymphopoietin
TRAIL: Tumor Necrosis Factor-Related Apoptosis-Inducing Ligand
uPA: Urokinase-Type Plasminogen Activator
UPLC-MS/MS: Ultra-Performance Liquid Chromatography-Tandem Mass Spectrometry
VEGF: Vascular Endothelial Growth Factor.
